# CMTM6: A Critical Prognostic Indicator in Non-Small Cell Lung Cancer

**DOI:** 10.7150/jca.93733

**Published:** 2024-03-04

**Authors:** Fang Dai, Yu-lian Duan, Qiang Feng, Shu-Ling Song, Ju-Lun Yang, Tao Lv

**Affiliations:** 1 College of Chemistry and Environmental Science, Qujing Normal University, Qujing, Yunnan, 655011, China.; 2 Graduate School, Kunming Medical University, Kunming, Yunnan, China.; 3 920th Hospital of the Joint Logistics Support Force of PLA, Kunming, Yunnan, China.; 4 College of Biological Resource and Food Engineering, Qujing Normal University, Qujing, Yunnan, 655011, China.

**Keywords:** CMTM6, prognosis, NSCLC, immunohistochemical

## Abstract

While CKLF-like MARVEL transmembrane domain containing 6 (CMTM6)'s role in stabilizing PD-L1 and immune evasion within tumors is established, its expression in lung cancer tissue and adjacent macrophages remains uncertain. The study aimed to elucidate this ambiguity by investigating CMTM6's role in non-small cell lung cancer (NSCLC) prognosis. Employing immunohistochemical staining on 141 NSCLC and 110 adjacent normal lung tissue samples, CMTM6 expression was evaluated using the HSCORE system. Interestingly, NSCLC exhibited significantly higher CMTM6 levels (161.04±86.60) compared to normal tissues (71.20±45.10) (*p* < 0.001), detected not only in cancer cells but also in macrophages, lymphocytes, and nearby bronchial epithelial cells. Stratifying patients by CMTM6 levels unveiled a correlation between heightened expression and poorer overall survival (*p* = 0.003), alongside a link to tumor-infiltrating lymphocytes (TIL) (*p* = 0.037), especially in cases with increased TIL. Multivariate analysis identified CMTM6 as an independent predictor of overall survival (*p* = 0.009), implying that elevated CMTM6 expression in NSCLC might signify an adverse prognostic marker for patient outcomes.

## Introduction

Lung cancer remains the deadliest tumor globally in terms of both illness and fatality rates 1. It encompasses two primary categories: small cell lung cancer (SCLC) and non-small cell lung cancer (NSCLC), with the latter constituting over 85% of cases. Among NSCLC, the primary histological types are adenocarcinoma and squamous cell carcinoma 2. Given the insidious onset of NSCLC, most tumors are diagnosed at an advanced stage. Traditional treatment modalities such as surgery, radiotherapy, and chemotherapy, while well-known, have limited applicability in advanced NSCLC and often result in severe side effects. In recent years, molecular targeted therapy has emerged as a promising approach for treating NSCLC. Several targeted drugs, including gefitinib targeting EGFR mutations, alectinib for ALK rearrangements, crizotinib for ROS1 rearrangements, and dabrafenib for BRAF 600E mutations, have been approved for clinical use. Despite these advancements, patients afflicted with NSCLC continue to experience a low five-year survival rate of only 17.4% 3, 4.

In recent years, significant progress has occurred in tumor immunotherapy, particularly in the development and marketing of PD1/PDL1 inhibitors. Although PD-1/PD-L1 immunological checkpoint inhibitors have displayed unprecedented durable responses in tumor therapy, the objective response is still limited, such as for pembrolizumab (PD-1 mAb) in the treatment of melanoma, where the progression-free survival time for 47.3% of patients is only 6 months. Atezolizumab (PD-L1 mAb) was found to have only 15% objective response in the treatment of renal clear cell carcinoma 5 and an 18% objective response in the treatment of NSCLC 6. The objective response to durvalumab (PD-L1 mAb) in treating urothelial carcinoma with high expression of PD-L1 was only 26.3%, while for urothelial carcinoma with low or no PD-L1 expression, the objective response was merely 4.1% 7. Additionally, PD-1 / PD-L1 immunological checkpoint inhibitor treatment has also shown efficacy in some PD-L1 negative patients 8. Thus, the clinical results have revealed that these commercially available PD-1/PD-L1 inhibitors have low response rates and the emergence of resistance 9. We hypothesized that there are other molecules linked to PD-1/PD-L1, affecting the inhibitory effect of PD-1/PD-L1 inhibitors.

Recently, two studies reported that PD-L1 was closely related to the CKLF-like MARVEL transmembrane domain containing family 6 (CMTM6). CMTM6 and PD-L1 colocalize on the tumor cell membrane where CMTM6 increases PD-L1 stability and mediates tumor immune escape 10, 11. It can be inferred that CMTM6 promotes PD-L1 expression and inhibits the activity of T cells, while the loss of CMTM6 restores T cell activity via relieving T cell immunosuppression. This suggests that CMTM6 may be a potential new target for tumor immunotherapy. CMTM6 is probably the cause of the low response rate to and drug resistance against PD-1/PD-L1 inhibitors 12. However, the results of these studies were derived from cell lines. While research has indicated a close correlation between aberrant CMTM6 expression and the prognosis of lung cancer tumors 13, 14, conflicting research outcomes exist. Therefore, expanding the scope of research with larger sample sizes becomes particularly crucial.

Here, this study selected 141 NSCLC patients and detected the expression of CMTM6 in NSCLC and adjacent normal lung tissue through tissue microarray and immunohistochemistry. The relationship between CMTM6 expression and clinical characteristics of NSCLC patients was analyzed to determine whether CMTM6 can be a potential prognostic factor for NSCLC.

## Materials and Methods

### Clinical samples

141 Patients with NSCLC who underwent radical surgical resection and pathological confirmation without preoperative chemotherapy or radiotherapy were selected from the 920th Hospital of the Joint Logistics Support Force of PLA from March 2014 to February 2018. Among them, there were 97 males and 44 females with an age range of 25-65 years and an average age of 57 years. Tumor tissue and adjacent normal tissues were obtained after surgery, and paraffin specimens were prepared by dehydration and dipping wax. The tumor specimens were pathologically diagnosed as adenocarcinoma or squamous cell carcinoma and staged depending on WHO Lung Cancer Tissue Type (2004) Standard typing. Follow-up data was collected by contacting the patient by phone or email. Overall survival was computed from the day of pathological diagnosis to the patient's death or last follow-up. The detailed clinicopathological features of 141 patients are shown in Table 1. Approval for our study was granted by the Research Ethics Committee of the 920th Hospital of the Joint Logistics Support Force of PLA.

### Tumor- infiltrating lymphocyte (TIL) determination

Lymphocyte infiltration determination was conducted following the method described by Huh et al 15. Pathological sections stained with H&E were observed under a microscope. The TIL amount was classified into 4 grades according to the degree of infiltration in the region with the most obvious tumor infiltration: Grade 0: No lymphocyte reaction; Grade 1: Scattered lymphocytes can be observed; Grade 2: moderate lymphocyte reaction or banded infiltration of lymphocytes can be observed; Grade 3: infiltration of large numbers of lymphocytes, disrupting the continuity of tumor cells. Subsequently, cases were categorized into a low TIL group (Grade 0-1) and a high TIL group (Grade 2-3) based on different infiltration conditions.

### Tissue microarrays

We reviewed all H&E-stained sections, identified representative areas, and marked them on a wax block. Then, a hollow tube with an internal diameter of 1.5 mm was used to pierce the tissue at each marker wax block and we made holes in the blank wax block (diameter 1.5 mm). The punched tissue cores were sequentially inserted into the holes of the blank wax block. After the tissue microarray wax blocks were completed the tissue microarray wax blocks were serially sliced at 4 µm to form microarray arrayed tissue chips.

### Immunohistochemistry analysis

Tissue sections were dewaxed in xylene for 10 minutes and then hydrated for 2 minutes in ethanol with decreasing concentrations (100%, 95%, 85% and 75% ethanol). The hydrated slides were placed in an autoclave containing pH 8.0 EDTA buffer for 5 minutes at 100 °C to facilitate antigen retrieval. Sections were incubated with 3% hydrogen peroxide for 10-15 minutes to block endogenous peroxidase activity. Following this, they were incubated overnight at 4°C in a refrigerator with the primary antibody (a polyclonal rabbit anti-human CMTM6 antibody) diluted in PBS (1:100; Sigma, St. Louis, USA). After removal of the primary antibody, the enzyme-labeled secondary antibody (Sheep anti-mouse/rabbit IgG antibody) working solution was added dropwise and incubated at 37°C temperature for 30 minutes. After each treatment, the slices were washed with PBS three times for 5 minutes. Upon removal of the secondary antibody, DAB color development and hematoxylin staining were performed.

CMTM6 immunoreactivity was evaluated using the HSCORE scoring system 16. It was assessed through a semi-quantitative immunoassay based on both the percentage of positive cells and the intensity of staining observed in these cells. The socring for section staining intensity was as follows:0 points for cells showing no staining; 1 point for weak staining (yellow); 2 points for moderate staining (moderate brownish yellow), and 3 points for strong staining (tan). The HSCORE values were calculated using the following formula: Hscore = (% of positive cells staining weakly×1) +(% of positive cells staining moderately×2) +(% of positive cells staining strongly×3). CMTM6 was primarily located in the cytomembrane and/or cytoplasm. The HSCORE was determined for each slide, with a maximum score of 300 and a minimum of 0.

### Statistical analysis

Categorical data were analyzed using SPSS version 18.0 software. All measurement data were presented as mean ± standard deviation, while count data was displayed as a percentage. The χ2 test was utilized to compare between groups. Kaplan-Meier analysis was employed to calculate survival rates, and log-rank test was used to assess statistical significance. Multivariate Cox proportional hazard models were employed for multivariate analysis to estimate the association between clinical features and overall survival. The CMTM6 cutoff value was determined using X-tile 3.6 software (Yale University) 17, 18.

## Results

### CMTM6 expression in NSCLC and adjacent normal tissue

**Figure 1:**
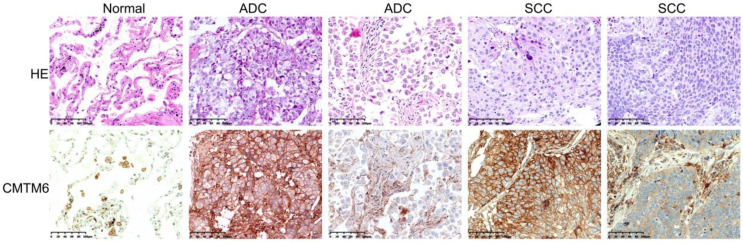
** Expression of CMTM6 was detected by immunohistochemistry (IHC) in NSCLC and adjacent normal tissue.** The expression of CMTM6 in lung adenocarcinoma (ADC) and squamous cell carcinoma (SCC) was higher than in adjacent normal tissue. Scale bar= 100 μM.

CMTM6 expression in cancerous and noncancerous tissues was analyzed through immunohistochemical staining using a rabbit anti-CMTM6 polyclonal antibody. CMTM6 was predominantly located in the cytomembrane and/or cytoplasm. While CMTM6 showed ubiquitous expression in NSCLC, some cases exhibited high expression, wherses others have low expression, assessed based on both the number of positive cells and the intensity of staining. Additionally, expression of CMTM6 was observed in macrophages, lymphocytes, and bronchial epithelial cells in adjacent normal lung tissues (Fig. 1). The Hscore was utilized to evaluate CMTM6 expression, yielding mean Hscore of 71.20±45.10 and 161.04± 86.60 in normal lung tissue and NSCLC, respectively (*P*<0.001). These findings significantly higher CMTM6 expression in NSCLC compared to adjacent normal tissues (Fig. 1).

### CMTM6 expression and its relationship to prognostic variables

**Figure 2:**
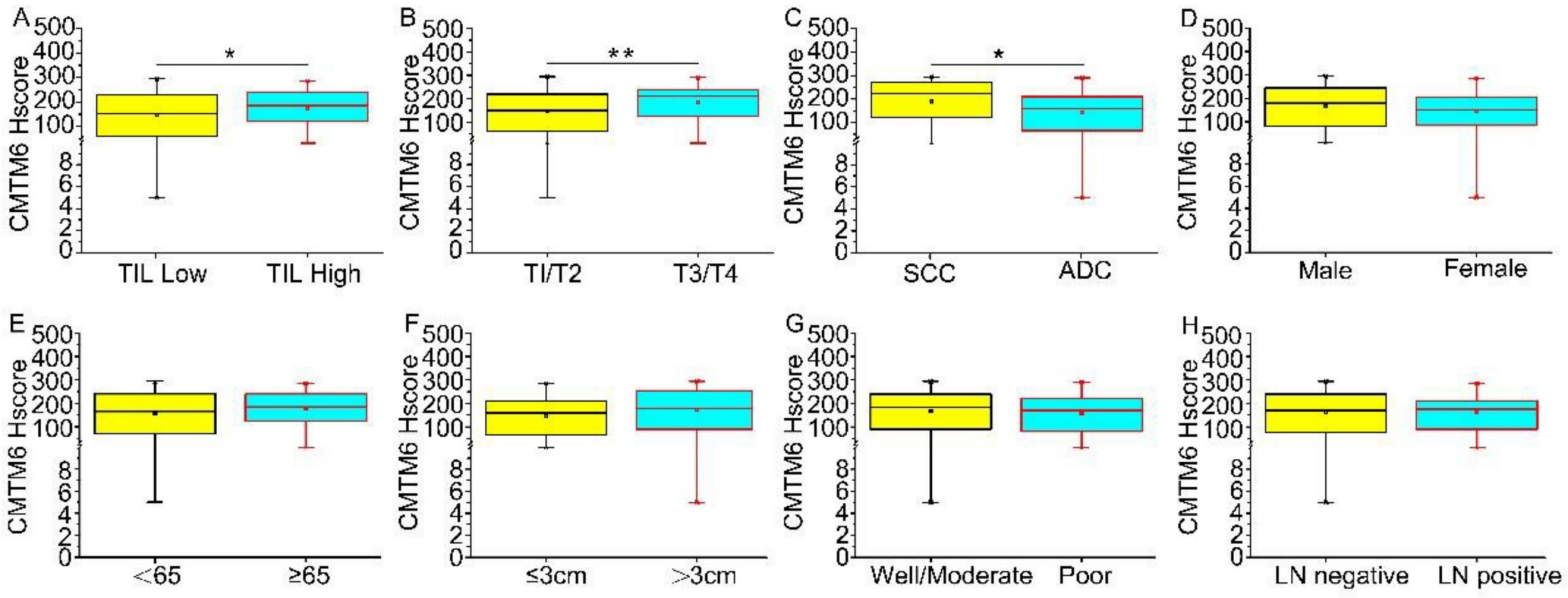
** The levels of CMTM6 expression across clinical characteristics (A-H) are illustrated.** Median CMTM6 scores and interquartile ranges are provided. (A) Tumor-infiltrating lymphocytes (TIL): Median levels of CMTM6 expression were higher in TIL high (median score 172.32 vs 146.68) compared to TIL low, with a *p*-value of 0.037. (B) T classification: the median levels of CMTM6 expression were higher in T3/T4 (median score 194.10 vs 152.48) compared to T1/T2, with a P-value of 0.010. (C)Tumor histology: Median levels of CMTM6 expression were higher in SCC (median score 189.24 vs 143.54) than in ADC, with a *p*-value of 0.021. (D-H) No statistically significant correlation was found between the expression level of CMTM6 and sex, age, tumor differentiation, or lymph node metastasis. Note: ADC, Adenocarcinoma; SCC, Squamous cell carcinoma; LN negative represents lymph nodes without cancer metastasis; LN positive represents lymph nodes with cancer metastasis. * *p*< 0.05, ** *p*< 0.01.

The optimal cutoff value for CMTM6 expression was assessed using X-tile plots based on patient survival time and the CMTM6 Hscore. An Hscore of 160 was identified as the optimal cutoff (*P*<0.01) (Extended Data Fig. 1). Utilizing this cutoff value, the study cases were divided into two groups: a high CMTM6 expression group (n=82) and a low expression group (n=59). Regarding tumor size, CMTM6 expression was notably higher in T3/4 compared to T1/2 (*p*=0.010). CMTM6 expression showed correlation with TIL (p=0.037) and was higher in cases with TIL. Furthermore, the level of CMTM6 expression exhibited associations with tumor histological types, specifically, squamous cell carcinoma displayed higher expression level compared to adenocarcinoma (*p*=0.021). However, no significant relationships were observed between the patient age, sex, lymph node metastasis, or tumor differentiation (Table 1 and Fig. 2).

### Univariate analysis of prognostic factors in NSCLC patients

**Figure 3:**
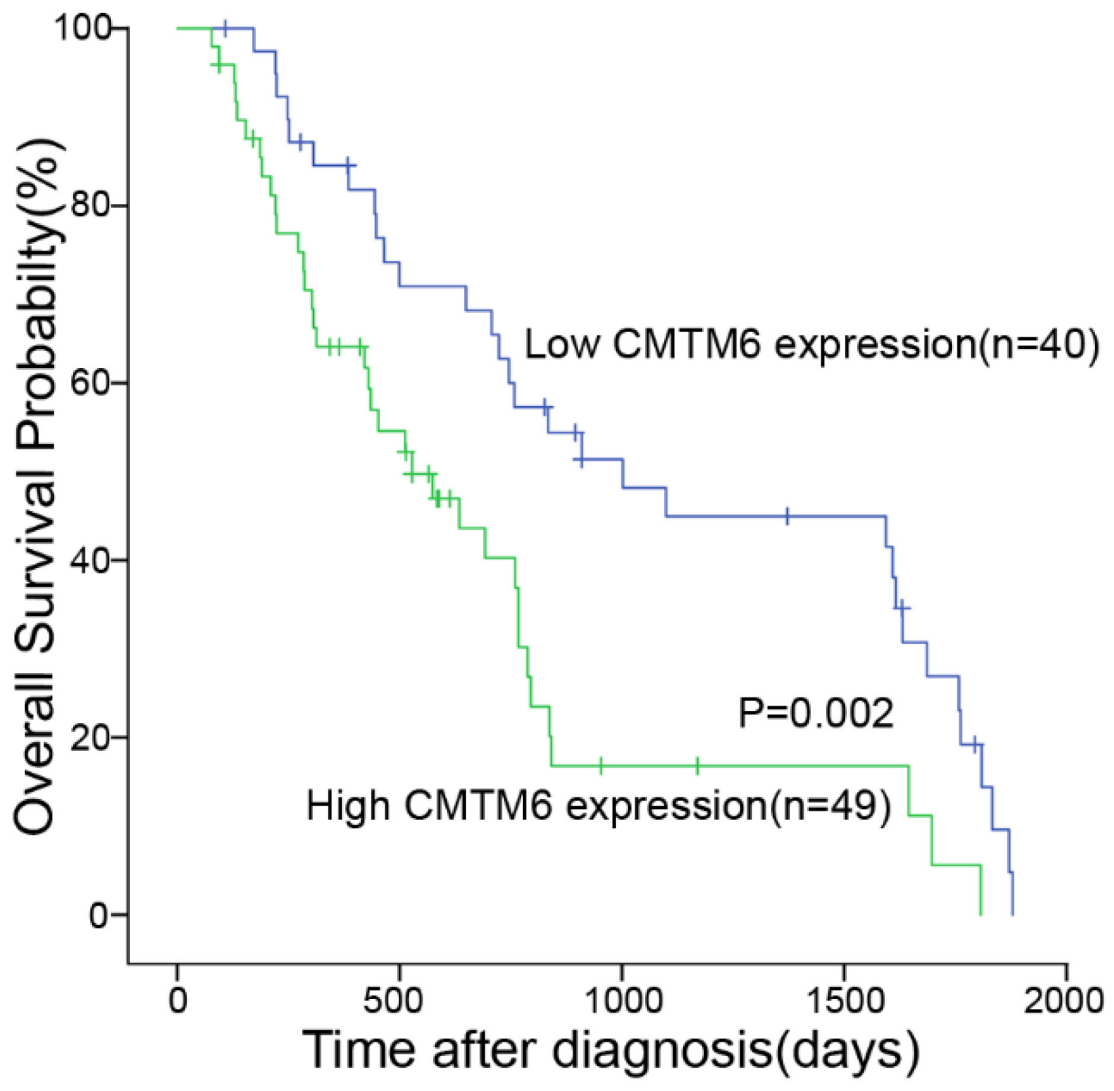
** High expression of CMTM6 was indicative of shorter overall survival in NSCLC patients.** Kaplan-Meier curves depicting CMTM6 expression in NSCLC patients in relation to their 5-year overall survival showed a statistically significant difference (log rank=9.159, *p* = 0.002).

We utilized the Kaplan-Meier test to assess the association between CMTM6 expression and the survival duration among the 89 patients. The analysis revealed correlations between CMTM6 expression, overall survival, and TIL (Table 2). These findings indicated a significant association where patients displaying high CMTM6 expression had notably shorter overall survival compared to those with low CMTM6 expression (*p*= 0.002, Fig. 3).

### Multivariate analysis of prognostic factors in NSCLC patients

Multivariate Cox proportional hazard models analysis showed that CMTM6 expression played a role as a predictor of a poor outcome regarding overall survival (*p* = 0.009) in NSCLC patients (Table 3).

## Discussion

CMTM6, initially cloned by Peking University's Center for Human Disease Genetics, encodes a transmembrane protein incorporating a MAL and related proteins for vesicle trafficking and membrane link (MARVEL) domain, critical for various biological processes like vesicle transport and tight junction formation. Abnormal expression or malfunctioning of this domain has been associated with diverse diseases 19. The extracellular loops within the MARVEL domain are crucial for binding to PD-L1 and CD58 20. The human CMTM family comprises nine members: CLLF and CMTM1-8 21. Most family members are dysregulated in tumors, serving as potential tumor suppressors affecting immune and male reproductive systems. While CMTM3, CMTM4, CMTM5, CMTM7, and CMTM8 are typically expressed in normal human tissues but downregulated or absent in many tumor cell lines, their decreased expression supports tumor cell proliferation and progression 22-26. However, unlike other family members, CMTM6 exhibits a 'cancer-promoting' effect by stabilizing PD-L1 molecules on tumor cell surfaces, consequently hindering the anticancer immune response of T cells.

Our study revealed heightened CMTM6 expression in NSCLC and its presence in macrophages, lymphocytes, and bronchial epithelial cells in adjacent normal lung tissues, indicating its role in tumor progression. Zhang S et al. also observed elevated CMTM6 levels in oral squamous cell carcinoma patients compared to adjacent normal tissues 27, while in Hela cells, increased CMTM6 expression spurred cell proliferation, potentially impacting cervical cancer 28. High CMTM6 expression in NSCLC correlated with lower overall survival rates, consistent with findings in malignant glioma patients 29. Although conflicting research on CMTM6 and lung cancer prognosis exists, our multivariate analyses identified CMTM6 expression as an independent predictor of overall survival, supported by an increased sample size aligning with Marian's animal experiments, reinforcing our research conclusions. Additionally, heightened CMTM6 expression corresponded to larger tumor sizes, especially in tumors exceeding 5 cm, suggesting its role in promoting tumor progression. CMTM6 expression varied among lung cancer histological types, notably more pronounced in squamous cell carcinomas than adenocarcinomas. Notably, CMTM6 expression lacked associations with age, sex, tumor differentiation, or lymph node metastasis. In summary, our findings indicate CMTM6's potential as a prognostic factor for NSCLC patients.

Elevated CMTM6 expression correlates with poor prognosis in NSCLC, likely fueling tumor progression by stabilizing PD-L1 on tumor cells. This interaction inhibits T cell activity upon PD-1/PD-L1 binding, dampening their function and evading immune attack, aiding cancer advancement. Targeting CMTM6 might offer potential in immunotherapy to counter this immune evasion mechanism. The tumor microenvironment plays a pivotal but dual role in lung cancer, capable of inhibiting or promoting tumor growth 30, 31. Comprising various elements like TIL, endothelial cells, and fibroblasts, TIL, particularly T cells, are crucial for effective immunotherapy and serve as prognostic markers 32-35. Our research reveals a link between CMTM6 expression and TIL abundance in NSCLC, suggesting CMTM6 involvement in immune responses. Intriguingly, despite increased TIL, inadequate T cell activation might hinder an effective anti-tumor immune response.

In summary, increased CMTM6 expression appears to contribute to NSCLC progression, potentially serving as a valuable prognostic indicator. As a novel biomarker, CMTM6 provides vital insights into the molecular mechanisms driving recurrence and metastasis in NSCLC, aiding in the identification of therapeutic targets for more effective targeted therapies.

## Figures and Tables

**Table 1:** Association between CMTM6 expression and clinicopathological variables of the studied NSCLC patients

Variables	No. of patients	CMTM6 Hscore	Expression of CMTM6		*p*
			High	Low	
Patients	141		82	59	
Age (median, years)	56.3 (34-73)				0.319
<65	114	156.74±88.45	64	50	
≥65	27	179.22±77.17	18	9	
Sex					0.091
Male	97	168.59±90.08	61	36	
Female	44	144.41±76.76	21	23	
Tumor histology					0.021
ADC	87	143.54±80.35	44	43	
SCC	54	189.24±89.56	38	16	
Tumor differentiation					0.852
w/m	69	165.93±90.98	40	29	
Poor	72	156.36±82.56	42	30	
T stage					0.010
T1/2	112	152.48±87.16	59	53	
T3/4	29	194.10±77.21	23	6	
Lymph node metastasis					0.237
Negative	84	159.44±90.66	46	38	
Positive	57	163.40±80.99	37	20	
TIL					
High	79	172.32±81.50	52	27	0.037
Low	62	146.68±91.35	30	32	

Note: ADC, Adenocarcinoma; SCC, Squamous cell carcinoma; TIL, Tumor-infiltrating lymphocytes; w/m, well/moderate differentiation.

**Table 2:** Univariate analysis using the Cox proportional hazards model to predict overall survival of NSCLC patients (89 patients)

Prognostic factors	*p*	HR	95% CI for HR	
			Lower	Upper
Age (years) (≥65 vs.<65)	0.204	1.527	0.794	2.938
Sex (female vs. male)	0.842	0.946	0.550	1.628
T classification (T3-4 vs. T1-2)	0.421	1.111	0.860	1.435
Tumor histology (ADC vs. SCC)	0.591	1.152	0.687	1.932
Tumor differentiation (poor vs. w/m)	0.356	1.258	0.773	2.049
Lymph node metastasis (P vs. N)	0.676	1.115	0.669	1.856
TIL (High vs. Low)	0.033	1.783	1.047	3.039
Expression of CMTM6 (High vs. Low)	0.003	2.191	1.304	3.682

Note: ADC, Adenocarcinoma; SCC, Squamous cell carcinoma; TIL, Tumor-infiltrating lymphocytes; w/m, well/moderate differentiation.

**Table 3:** Multivariate analysis using the Cox proportional hazards model to predict overall survival of lung cancer patients (89 patients)

Prognostic factors	*p*	HR	95% CI for HR	
			Lower	Upper
Age (years) (≥65 vs.<65)	0.466	1.305	0.638	2.669
Sex (female vs. male)	0.235	1.555	0.751	3.218
T classification (T3-4 vs. T1-2)	0.625	1.137	0.679	1.905
Tumor histology (ADC vs. SCC)	0.426	1.354	0.642	2.853
Tumor differentiation (poor vs. w/m)	0.989	1.004	0.541	1.863
Lymph node metastasis (P vs. N)	0.419	0.798	0.461	1.380
TIL (High vs. Low)	0.094	1.635	0.919	2.907
Expression of CMTM6 (High vs. Low)	0.009	2.370	1.236	4.546

Note: ADC, Adenocarcinoma; SCC, Squamous cell carcinoma; TIL, Tumor-infiltrating lymphocytes; w/m, well/moderate differentiation.

## Abbreviations

NSCLC: non-small cell lung cancer
EGFR: epidermal growth factor receptor
ALK: anaplastic lymphoma kinase
ROS1: ROS proto-oncogene 1, receptor tyrosine kinase
BRAF: B-Raf proto-oncogene, serine/threonine kinase
PD1: Programmed Death 1
PDL1: Programmed Death-Ligand 1
